# Potential risk of disease modifying therapies on neoplasm development and coadjutant factors in multiple sclerosis outpatients

**DOI:** 10.1038/s41598-021-91912-x

**Published:** 2021-06-15

**Authors:** Rosalía Gil-Bernal, Juan Luis González-Caballero, Raúl Espinosa-Rosso, Carmen Gómez-Gómez

**Affiliations:** 1 Department of Biochemistry, Faculty of Medicine, University of Cadiz, Plaza Fragela, s/n, 11003 Cadiz, Spain; 2 Department of Statistics and Operations Research, Faculty of Medicine, University of Cadiz, Cadiz, Spain; 3 Department of Neurology, Hospital Universitario Puerta del Mar (HUPM), Cadiz, Spain

**Keywords:** Neurology, Risk factors

## Abstract

Neoplasm development in Multiple Sclerosis (MS) patients treated with disease-modifying therapies (DMTs) has been widely discussed. The aim of this work is to determine neoplasm frequency, relationship with the prescription pattern of DMTs, and influence of the patients’ baseline characteristics. Data from 250 MS outpatients were collected during the period 1981–2019 from the medical records of the Neurology Service of the HUPM (*Hospital Universitario Puerta del Mar*)—in Southern Spain—and analysed using Cox models. Neoplasm prevalence was 24%, mainly located on the skin, with cancer prevalence as expected for MS (6.8%). Latency period from MS onset to neoplasm diagnosis was 10.4 ± 6.9 years (median 9.30 [0.9–30.5]). During the observation period β-IFN (70.4% of patients), glatiramer acetate (30.4%), natalizumab (16.8%), fingolimod (24.8%), dimethyl fumarate (24.0%), alemtuzumab (6.0%), and teriflunomide (4.8%) were administered as monotherapy. Change of pattern in step therapy was significantly different in cancer patients vs unaffected individuals (*p* = 0.011) (29.4% did not receive DMTs [*p* = 0.000]). Extended Cox model: Smoking (HR = 3.938, CI 95% 1.392–11.140, *p* = 0.010), being female (HR = 2.006, 1.070–3.760, *p* = 0.030), and age at MS diagnosis (AGE-DG) (HR = 1.036, 1.012–1.061, *p* = 0.004) were risk factors for neoplasm development. Secondary progressive MS (SPMS) phenotype (HR = 0.179, 0.042–0.764, *p* = 0.020) and treatment-time with IFN (HR = 0.923, 0.873–0.977, *p* = 0.006) or DMF (HR = 0.725, 0.507–1.036, *p* = 0.077) were protective factors. Tobacco and IFN lost their negative/positive influence as survival time increased. Cox PH model: Tobacco/AGE-DG interaction was a risk factor for cancer (HR = 1.099, 1.001–1.208, *p* = 0.049), followed by FLM treatment-time (HR = 1.219, 0.979–1.517). In conclusion, smoking, female sex, and AGE-DG were risk factors, and SPMS and IFN treatment-time were protective factors for neoplasm development; smoking/AGE-DG interaction was the main cancer risk factor.

## Background

Although survival after the onset of Multiple Sclerosis (MS) has historically increased for 17 years, as reflected in the first works 41 years ago, patient life expectancy is, on average, 7 years below the general population data^1^. While there are different estimations about the increased risk for all-cause mortality (up to threefold), everyone agrees that MS itself is the main cause of death^2,3^.

Disease-modifying therapies (DMTs) have been the most important advance in MS treatment, from the so-called first-generation drugs, β-interferon (IFN) and glatiramer acetate (GA), approved in the mid-1990s, until the introduction of the latest drugs in the 2010s, such as fingolimod (FLM), alemtuzumab (ALB), dimethyl fumarate (DMF), teriflunomide (TRF), or the most recently approved purine analogue, cladribine^4^. Despite their benefits, undesirable effects are expected, especially in relation to infection and malignancy^5^. IFN liver failure or progressive multifocal leukoencephalopathy from natalizumab (NTB), although not frequent, are seen as possible threats in routine clinical practice, unlike the development of neoplasms, a rare but dangerous side effect.

In the literature, we found a disparity in the data for both neoplasm frequency in MS patients and the role of DMTs in the neoplasm development, from an increase in cancer-related deaths (1.9-fold)^6^ to normal or decreased cancer prevalence, although the hazard risk depends on the type of tumour^7,8^. Increased cancer risk has been observed among patients treated with IFN, GA, NTB, and ALB^9,10^. The development of skin cancer is contemplated in the technical information about GA, FLM (basal cell and squamous cell skin cancers, Bowen’s disease, melanoma, Kaposi’s sarcoma), and ALB (papilloma) (available at http://www.ema.europa.eu, European Medicines Agency, EMA, last accesed May 2021).

Therefore, the aim of this work is to investigate the therapeutic pattern followed and its relationship with the incidence of neoplasia in MS outpatients. We hypothesise that DMTs could constitute risk factors for the development of neoplasia. Patients’ baseline characteristics were considered for their possible contribution to the development of neoplasms.

## Methods

### Patient recruitment

This retrospective study was based on outpatients from the Department of Neurology at the Hospital Universitario Puerta del Mar (HUPM), attended to in consultation from onset of MS between 1981 and 2019. According to the Spanish Statistical Office, the area served by HUPM had an average population of 220 thousand during this period (data available at  http://www.ine.es, *Instituto Nacional de Estadística, INE*). The data source was the digital medical record used by neurologists conducting a multidisciplinary team. Other independently-trained team members carried out data extraction and statistical analysis. Individuals previously suffering from a neoplasm or diagnosed with Clinically Isolated Syndrome were excluded. As a result, 314 consecutive cases were analysed, 250 of which fulfilled the inclusion criteria. The last case was diagnosed in September 2017, and the follow-up of patients ended on the last database registered individual (March 31, 2019).

### Measures

The analysed variables were: (a) demographic and baseline characteristics: sex, age at MS onset (AGE-DG); disease phenotype; as possible contributing factors, neoplasm family history and smoking; (b) factors related to neoplasm development: presence and number of neoplasms; malignancy/benignity; tumour lineage; age at neoplasm onset; latency period from the start of DMTs until neoplasm appearance c) factors related to the medication received: type and number of drugs; use order; treatment time for each drug; length of treatment.

### Statistical analysis

Categorical variables were expressed as number and percentage of observed data; numeric variables were represented as mean ± standard deviation and median (minimum–maximum). Association between categorical variables was contrasted by χ^2^ test, or if these conditions were not verified, by Fisher’s exact test. Quantitative variable comparisons were performed using the Student’s t-test. Associations were considered significant when *p* < 0.05. For each patient, the observation period (survival time) started with MS diagnosis until neoplasm appearance or the end of the study (censored case).

Several covariate analyses were performed to estimate the role of DMTs and other possible contributing factors to neoplasm development. The Cox proportional hazard model was used to analyse the predictors associated with the hazard rate (HR), with a 95% confidence interval (CI). For assessing the proportional hazard (PH) for each predictor of interest, we also estimated *p*-values between the ranked survival and the residuals. When the predictors did not satisfy the PH assumption, an extended Cox model was used. For this purpose, we included time-dependent variables to measure the interaction factor with time exposure^11^. The data were processed using IBM SPSS Statistics 24 and Epidat 3.1 software.

### Ethical considerations

The study was approved by the *Comité de Ética de la Investigación de Cádiz. Servicio Andaluz de Salud. Consejería de Salud. Junta de Andalucía.* HUPM. Av. Ana de Viya 21, 11009 CÁDIZ (SPAIN) (phone + 0034 956002100) (ceic.hpm.sspa@juntadeandalucia.es). The need of the informed consent was waived by the *Comité de Ética de la Investigación de Cádiz*. All the experiments were carried out in accordance with the relevant guidelines and regulations.

## Results

### Patient baseline features

Patient baseline features were registered in Table 1. Women accounted for 63.2% (n = 158) of the individuals. Mean AGE-DG was 34.0 ± 11.3 years (median 32.0 [13–71)], mostly ranging from 20–40 years (62.4%, n = 156). Relapsing–remitting variant (RRMS) (83.2%, n = 208) was the predominant medical condition. Neoplasm family history was present in 10.4% (n = 26) and tobacco consumption in 44% (n = 110) of individuals. In particular, 24% (n = 60) developed some kind of neoplasm, alone or successively. Five patients suffered from a second (n = 4) or a third process (n = 1). Out of the sampled patients, 6.8% (n = 17, 28.3% of neoplastic patients) suffered malignancy and two of these individuals presented benign tumour. Mean age at tumour diagnosis was 46.2 ± 11.3 years (median 45.5 [26–80]) for neoplasm and 52.1 ± 8.4 for malignancy (median 53.0 [37–69]). Feminine gender, smoking, and family history were significantly present in neoplasm patients as compared to patients who did not present these characteristics (*p* < 0.05). Cancer patients were older at AGE-DG (39.8 ± 12.3 years, median 40 [21–64)] than the remaining individuals (33.58 ± 11.12, median 32.0 [13–71], n = 233) (mean (*p* = 0.027), median (*p* = 0.043)).

**Table 1:** Patient baseline features.

	TOTAL** (N = 250)	NEO (n = 60)	NNEO (n = 190)	p*	CANCER (n = 17)	NCANCER (n = 233)	p*
**Woman, n(%)**	158 (63.2)	46 (76.7)	112 (58.9)	0.013	12 (70.6)	146 (62.7)	0.513
**Age at onset**							
Mean ± SD	34.0 ± 11.3	35.6 ± 11.5	33.5 ± 11.2	0.205	39.8 ± 12.3	33.58 ± 11.12	0.027
Median (range)	32.0 (13–71)	34.0 (18–71)	32.0 (13–67)	0.271	40 (21–64)	32.0 (13–71)	0.043
Age Intervals, n(%)				0.264			0.085
< 20 years	21 (8.4)	2 (3.3)	19 (10.0)		0 (0.0)	21 (9.0)	
20–40 years	156 (62.4)	40 (66.7)	116 (61.1)		9 (52.9)	147 (63.1)	
> 40 years	73 (29.2)	18 (30.0)	55 (28.9)		8 (47.1)	65 (27.9)	
**MS phenotype, n(%) §**				0.286			0.297
RRMS	208 (83.2)	51 (85.0)	157 (82.7)		12 (70.5)	196 (84.1)	
PPMS	19 (7.6)	6 (10.0)	13 (6.8)		2 (11.8)	17 (7.3)	
SPMS	21 (8.4)	2 (3.3)	19 (10)		2 (11.8)	19 (8.2)	
RPMS	2 (0.8)	1 (1.7)	1 (0.5)		1 (5.9)	1 (0.4)	
**Smoking, n(%)**	110 (44)	35 (58.3)	75 (39.5)	0.010	9 (52.9)	75 (39.5)	0.442
**Neo family history, n(%)**	26 (10.4)	11 (18.3)	15 (7.9)	0.021	2 (11.8)	24 (10.3)	0.849
**Average follow-up time (years)**^**¥**^							
Mean ± SD	12.1 ± 7.6	10.4 ± 6.9	12.8 ± 7.7	0.031	12.2 ± 7.6	12.2 ± 7.6	0.992
Median (range)	11.2 (0.9–37.5)	9.4 (0.9–30.5)	11.6 (1.3–37.5)	0.024	9,9 (2.3–27.8)	11.2 (0.9–37.5)	0.972

Comparison of individuals affected vs unaffected by neoplasia.

*p value from the χ-test or T-test.

^§^ RRMS: relapsing–remitting MS; PPMS: primary progressive MS; SPMS: secondary progressive MS; PRMS: progressive-relapsing MS.

^¥^Time from MS diagnosis to first neoplasm/cancer appearance or end of the observation period (censored cases).

**TOTAL: whole sample; NEO: patients with neoplasm; NNEO: neoplasm-free patients; CANCER: patients with cancer; NCANCER: cancer-free patients.

Mean MS duration was 13.1 ± 7.8 years. Table 1 shows the observation periods. The latency period from MS to neoplasm appearance was 10.4 ± 6.9 years (median 9.30 [0.9–30.5]), significantly less than the observation period for censured cases (mean 12.8 ± 7.7, median 11.6 [1.3–37.5]) (mean *p* = 0.031, median *p* = 0.024).

### Tumour lineage

Table 2 indicates tumour frequency, site, developmental lineage, and cell line. Sixty patients developed some kind of neoplasm (n = 66), 74.2% were benign (n = 49), with melanocytic nevus (18.2% of neoplasms, n = 12) and uterine myoma (12.1%, n = 8) being most common. The most frequent locations were cutaneous tissue (keratosis, melanocytic nevus, melanoma, basal cell carcinoma) (37.9%, n = 25) and the myometrium (12.1%, n = 8). The neoplasms originated in the ectoderm (65.2%, n = 43), mesoderm (27.3%, n = 18), or had a mixed origin (7.5%, n = 5). The epithelial cell line was most frequent (38.3% of neoplasm, n = 27), followed by the melanocytic line (21.2%, n = 14). Fourteen malignant tumours were carcinomas.

**Table 2:** Neoplasm types and frequency.

**Neoplasm type (n=66)**	Frecuency n (%)	Cell line	Embryogenic lineage
**BENIGN** (n = 49, 74.2%)			
Pleomorphic parotid adenoma	1 (1.5)	Mixed	Mixed
Hürtle cell thyroid adenoma	1 (1.5)	Epithelial	Ectodermal
Liver angioma	1 (1.5)	Blood vessel	Mesodermal
Renal angiomyolipoma	1 (1.5)	Mixed	Mixed
Breast fibroadenoma	3 (4.5)	Mixed	Mixed
Fibroma	2 (3.0)	Connective	Mesodermal
MGUS§	1 (1.5)	Hematopoietic	Mesodermal
Ganglion (interphalangeal)	1 (1.5)	Connective	Mesodermal
Benign prostatic hyperplasia	1 (1.5)	Epithelial	Ectodermal
Lipoma	2 (3.0)	Adipose tissue	Mesodermal
Cerebral meningioma	2 (3.0)	Meninges	Mesodermal
Uterine myoma	8 (12.1)	Myometrium	Mesodermal
Acoustic neurilenoma	1 (1.5)	Nervous tissue	Ectodermal
Brachial plexus neurilenoma	1 (1.5)	Nervous tissue	Ectodermal
Common melanocytic nevus	12 (18.2)	Melanocytes	Ectodermal
Uterine endocervical polyp	1 (1.5)	Epithelial	Ectodermal
Hyperplastic colonic polyp	1 (1.5)	Epithelial	Ectodermal
Laryngeal polyp	1 (1.5)	Epithelial	Ectodermal
Gallbladder benign polyp	1 (1.5)	Epithelial	Ectodermal
Vocal polyp	1 (1.5)	Epithelial	Ectodermal
Actinic keratosis	1 (1.5)	Epithelial	Ectodermal
Seborrheic keratosis	5 (7.6)	Epithelial	Ectodermal
**MALIGNANT** (n = 17, 25.8%)			
Breast adenocarcinoma	2 (3.0)	Epithelial	Ectodermal
Prostate Adenocarcinoma	2 (3.0)	Epithelial	Ectodermal
Lung adenocarcinoma	2 (3.0)	Epithelial	Ectodermal
Basal-cell carcinoma	5 (7.6)	Epithelial	Ectodermal
Papillary thyroid carcinoma	2 (3.0)	Epithelial	Ectodermal
Ovarian serous Carcinoma	1 (1.5)	Epithelial	Ectodermal
Melanoma	2 (3.0)	Melanocytes	Ectodermal
Chronic myeloid leukaemia	1 (1.5)	Hematopoietic	Mesodermal

^§^MGUS: Monoclonal gammopathy of uncertain significance.

### DMT pattern and neoplasm development

A total of 92.9% (n = 232) of individuals received DMTs, 41.4% (n = 96) of which continued on their first therapy, while 58.6% (n = 136) was required to switch drugs due to inefficacy or non-compliance. According to clinical criteria, 18 patients remained untreated (mean AGE-DG 47.1 ± 11.0, median 48.5 [29–67]; mean age at neoplasm diagnosis was 62.2 ± 5.1, median 62.5 [54–69]; 44.4% women). The drugs administered as monotherapy were IFN (n = 176, 70.4% of patients), GA (n = 76, 30.4%), NTB (n = 42, 16.8%), FLM (n = 62, 24.8%), DMF (n = 60, 24.0%), TRF (n = 12, 4.8%), and ALB (n = 15, 6.0) (Table 3).

**Table 3:** Differences in therapy pattern between neoplasm-affected and unaffected patients.

**Drug n (%)**	Total n = 250	NEO (n = 60)	No NEO (n = 190)	*p*	CANCER (n = 17)	No CANCER (N = 233)	*p*
None	18 (7.2)	6 (10)	12 (6.3)	0.336	5 (29.4)	13 (5.6)	0.000
INF	176 (70.4)	42 (70)	134 (70.5)	0.938	10 (58.8)	166 (71.2)	0.279
GA	76 (30.4)	23 (38.3)	53 (27.9)	0.125	4 (23.5)	72 (30.9)	0.524
NTB	42 (16.8)	9 (15.0)	33 (17.4)	0.669	2 (11.8)	40 (17.2)	0.565
FLM	62 (24.8)	15 (25.0)	47 (24.7)	0.967	5 (29.4)	57 (24.5)	0.648
DMF	60 (24.0)	9 (15.0)	51 (26.8)	0.061	3 (17.6)	57 (24.5)	0.525
TRF	12 (4.8)	4 (6.7)	8 (4.2)	0.438	0 (0.0)	12 (5.2)	0.338
ALB	15 (6.0)	2 (3.3)	13 (6.8)	0.318	1 (5.9)	14 (6.0)	1.000
**No. of drugs administered, n (%)**				0.813			0.011
0	18 (7.2)	6 (10)	12 (6.3)		5 (29.4)	13 (5.6)	
1	96 (38.4)	22 (36.6)	74 (38.9)		3 (17.6)	92 (39.5)	
2	82 (32.8)	19 (31.7)	63 (33.2)		7 (41.2)	76 (32.6)	
> 2	54 (21.6)	13 (21.7)	41 (21.6)		2 (11.8)	52 (22.3)	
**Treatment length (years)**							
Mean ± SD	8.2 ± 5.6	8.8 ± 6.0	8.1 ± 5.5	0.500	8.3 ± 6.9	8.2 ± 5.5	0.865
Median (range)	8.3 (0.0–22.8)	8.7 (0.0–22.8)	8.0 (0.0–22.4)	0.412	8.9 (0.0–22.0)	8.3 (0.0–22.8)	0.977

Treatment length of each drug (years)							
		Mean ± SD	Median (range)				
	IFN	4.6 ± 5.6	1.8 (0.0–22.0)				
	GA	1.5 ± 3.1	0.0 (0.0–13.3)				
	NTZ	0.7 ± 2.0	0.0 (0.0–9.8)				
	FLM	0.8 ± 1.6	0.0 (0.0–7.5)				
	DMF	0.5 ± 1.0	0.0 (0.0–3.7)				
	TLF	0.1 ± 0.5	0.0 (0.0–3.6)				
	ALB	0.1 ± 0.5	0.0 (0.0–3.0)				

Drug prescribing frequency in the successive changes on sequential therapy							
	At 1st	At 2nd	At 3rd	At 4th	At 5th		
	n (%) (referred to whole sample [n = 250])						
IFN	162 (64.8)	14 (5.6)	4 (1.6)	0	0		
GA	38 (15.2)	37 (14.8)	0	1 (0.4)	0		
NTB	7 (2.8)	23 (9.2)	10 (4.0)	2 (0.8)	0		
FLM	6 (2.4)	28 (11.2)	25 (10.0)	3 (1.2)	0		
DMF	14 (5.6)	29 (11.6)	9 (3.6)	8 (3.2)	0		
TRF	2 (0.8)	5 (2.0)	4 (1.6)	1 (0,4)	0		
ALB	3 (1.2)	1 (0.4)	3 (1.2)	7 (2.8)	1 (0.4)		

For the case of neoplastic patients, 10% did not receive treatment, while 36.7% (n = 22) were being treated with their first drug, 31.7% (n = 19) with a second drug, and 13 (21.7%) with a third, fourth, or fifth option, with no differences with respect to non-neoplastic patients. A total of 29.4% (n = 5) of cancer patients did not receive treatment vs cancer-free individuals (5.1%, n = 13) (*p* = 000), with differences between both groups also observed in the escalating therapy (*p* = 0.011) (Table 3).

Treatment duration, overall and specifically for each drug, is recorded in Table 3. Median treatment length was around eight years in all groups (whole sample 8.2 ± 5.6 years, median 8.3 [0.0–22.8]). IFN therapy had the longest duration of treatment (mean 4.6 ± 5.6 years, median 1.8 [0.0–22.0)]. Mean treatment time with DMF resulted significantly lower in neoplasm patients (mean 0.3 ± 0.7, median 0.0 [0–3.7]) vs unaffected individuals (mean 0.6 ± 1.1, median 0.0 [0.0–3.7]) (mean (*p* = 0.005), median (*p* = 0.43)).

**Figure 1:**
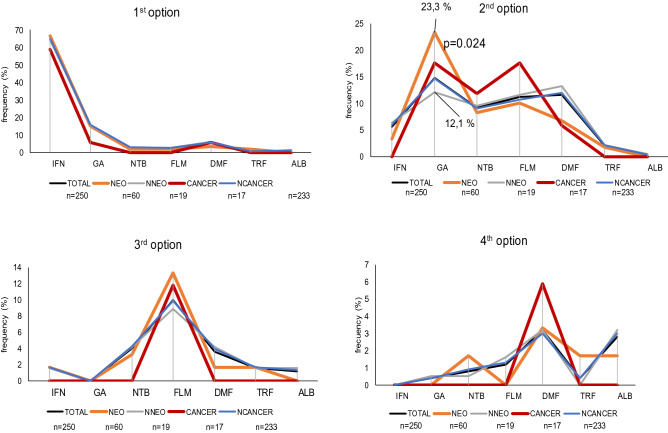
Drug prescription frequency in the therapeutic sequence. Percentages were calculated in relation to the group total cases.

Figure 1 shows the drug prescription frequency in sequential therapy for the whole sample (detailed in Table 3) as well as the four groups studied (NEO: patients with neoplasm, NNEO: neoplasm-free patients; CANCER: patients with cancer; NCANCER: cancer-free patients). IFN was the main first choice in all groups (64.8%, n = 162). As a second option, a wider range of drugs was prescribed, with GA being used significantly more in neoplastic (23.3%, n = 14) as compared to “healthy” patients (12.1%, n = 23) (*p* = 0.024). Individuals who received IFN as a third option had previously received it as a first option. Only one patient was required to change to a fifth drug (in the following order: IFN, GA, FLM, DMF, ALB) during the observation period and developed malignancy.

### Cox proportional hazard risk (HR) models to evaluate the contribution of variables to neoplasm development

Variables with statistical significance (*p* < 0.05) or no significance but with possible long-term influence (*p* < 0.15), estimated by the Wald test, are recorded in Table 4. The PH hypothesis was not admissible for smoking (TAB) (*p*(PH) = 0.046), duration of IFN use (TIFN) (*p*(PH) = 0.002), and covariates with *p*(PH)-values between 0.662–0.986. This was solved using an extended Cox model, including two time-dependent covariates: a) survival time (ST) multiplied by TAB (STxTAB) and b) ST multiplied by TINF-r (STxTINF-r) (TIFN was previously categorised as TINF-r = 0 and TINF-r > 0). HR values were constant throughout the follow-up period, except for variables TAB and TINF.

**Table 4:** Cox analysis to assess the risk of neoplasm development.

	Coef	SE	Wald	*p*	HR	95% CI for HR	
**Extended Cox model to evaluate influence of covariates on the of the first neoplasm appearance**							
AGE-DG	0.035	0.012	8.417	0.004	1.036	1.012–1.061	
GEN	0.696	0.321	4.718	0.030	2.006	1.070–3.760	
TAB	1.371	0.531	6.675	0.010	3.938	1.392–11.140	
HYS	0.537	0.354	2.302	0.129	1.711	0.855–3.426	
SPMS	-1.723	0.742	5.396	0.020	0.179	0.042–0.764	
TINF	-0.080	0.029	7.682	0.006	0.923	0.873–0.977	
TDMF	-0.322	0.182	3.119	0.077	0.725	0.507–1.036	
ST x TAB	-0.076	0.045	2.930	0.087	0.926	0.849–1.011	
ST x TINF	0.053	0.033	2.667	0.102	1.055	0.989–1.124	
Sample size = 250; − 2LnLikelihood = 524.849; LR (M1-M0) = 45.849 (9df) ; *p* = 0.000							

	Coef	SE	Wald	*p*	HR	95% CI for HR	*p*(PH)*
**Cox regression model to estimate influence of covariates in malignancy**							
AGE-DG	0.055	0.029	3.626	0.057	1.056	0.998–1.117	0.129
TAB	-3.228	2.008	2.586	0.108	0.040	0.001–2.027	0.849
TFLM	0.198	0.112	3.126	0.077	1.219	0.979–1.517	0.133
AGE-DG x TAB	0.095	0.048	3.885	0.049	1.099	1.001–1.208	0.675
Sample size = 250; − 2LnLikelihood = 133.865; LR (M1-M0) = 22.411 (9df) ; *p* = 0.000							

AGE-DG: age at MD diagnosis; GEN: gender; TAB: smoking; HYS: family history of neoplasm; SPMS: secondary progresive MS phenotype; TINF: time of INF use; TDMF: time of DMF use; TFLM: time of FLM use; ST: survival time (neoplasm-free time).

*We have included non-significant *p* (PH)-values to contrast each covariate.

Using this model (Table 4), we found that for each increasing year of MS diagnosis, risk increases 3.6% (HR = 1.036, CI95% 1.012–1.061, *p* = 0.004), women have twice the risk than men (HR = 2.006, CI95% 1.070–3.760, *p* = 0.030), and smoking quadruples the risk of development of a first neoplasm (HR = 3.938, CI95% 1.392–11.140, *p* = 0.010). To a lesser extent, family history of neoplasm increased risk 1.7 times (HR = 1.711, CI95% 0.855–3.426, *p* = 0.0129). Secondary progressive MS (SPMS) was found to be a protective risk factor with ≈1/5 the risk of neoplasm development with respect to other MS phenotypes (HR = 0.179, CI95% 0.042–0.764, *p* = 0.020).

TINF (HR = 0.923, CI95% 0.873–0.977, *p* = 0.006) or duration of DMF use (TDMF) (HR = 0.725, CI95% 0.507–1.036, *p* = 0.077) acted as risk protectors in such a way that for each treatment year with IFM or DMF, neoplasm risk decreased by 8% or 27%, respectively. STxTAB (HR = 0.926, CI95% 0.849–1.011, *p* = 0.087) and STxTINF (HR = 1.055, CI95% 0.989–1.124, *p* = 0.102), shown as survival time (free-neoplasm time), modified the predicted risks so that with each passing year, both smoking risk and protective nature of TIFN decreased.

A second Cox proportional HR model analysed the predictors for cancer-free time (Table 4). AGE-DG (HR = 1.056, CI95% 0.998–1.117, *p* = 0.057) and TAB (HR = 0.040, CI95% 0.001–2.027, *p* = 0.108) influenced malignancy. Increased age at diagnosis raised the risk, while smoking appeared to be a protective factor. However, when variable-interaction effect was estimated (HR = 1.099, CI95% 1.001–1.208, *p* = 0.049), AGE-DG, as a risk factor, increased in smokers and TAB increased cancer risk as AGE-DG increased. Thus, smoking becomes a risk factor for patients over the sampled mean age (34 years). Note that at this age, the HR of TAB + AGE-DG TAB is exp(− 3.228 + 34 * 0.095) = 1.002. Finally, duration of FLM use was a risk factor (HR = 1.219, CI95% 0.979–1.517, *p* = 0.077) for cancer development.

## Discussion

Are detected frequency and malignancy type similar to that of the general population? In the European population^12^, breast (13.5% of all cancer cases), prostate (12.1%), and lung cancer (11.9%) represent 37.5% of all tumours, in a similar proportion to our data (35.3% of cancers, n = 7). However, in our sample, skin melanoma represented 11.8% (2 of 17), while, in Europe, it is the sixth most frequent cancer (3%); although non-melanoma skin cancer (particularly basal cell carcinomas) data were similar in sampled individuals (71.4% of cutaneous cancers) vs the general population (70–80%).

We detected 21 types of benign neoplasms, many of them common in individuals over the age of 50: MEGUS in 1% > 50; Hürtle-cell adenoma of the thyroid in 0.5–1% of adults; hyperplastic colonic polyps in 30% of adults, 50% in the elderly^13^. Uterine myoma was the second most frequent neoplasm in the patients sampled, in consonance with myometrial tumours representing 20% of benign tumours in women (in our sample, 23.3% [n = 8] of 37 women suffered from benign processes)^14^.

Several studies suggested an increased risk of breast and central nervous system cancer, or benign neoplasm (meningioma, adenoma), but not especially skin cancer^7,9,15^. On the contrary, other works detected a decreased HR^16^: intense MS immune activity or immunomodulatory treatment has been hypothesised as an explanation^17^. In this sense, the Cox analysis showed that increased age at MS onset implied a greater risk of both neoplasm and malignancy. Would this be due to a “protective” effect of the MS treatment, or to senility itself? Untreated patients were older; both at MS onset and cancer diagnosis, and 27.8% developed cancer, a higher value than the 13.92% for a Spanish population ≥ 65 under multi-morbidity conditions^18^.

Moreover, a population-based study (51% women; mean age 47.76 ± 10.99, 77% population > 24 years old) reported a prevalence of 3%^19^. Despite this, cancer prevalence in our sample (6.8%) was in the range of 2.6–7.3% recorded in the MS literature^10,16,20^. Neoplasm type and relative frequency were those expected in the general population, except for skin tumours (37.9%, 25 of 66 of neoplasms diagnosed), being the most prevalent. This is an expected side-effect of DMTs, recorded in the *adverse reactions* section of the *Summary of Product Characteristics* for some drugs such as FLM, as mentioned above, or GA: ≥ 1/100 to < 1/10 of GA-treated patients could develop benign skin neoplasms; ≥ 1/1000 to < 1/10 could develop skin cancer. Technical information on other more recently introduced active substances such as NTB (00’) and ALB (10’s) includes a recommendation for additional monitoring for any suspicious reaction detected (http://www.ema.europa.eu), but this is rather directed at other health-compromising side-effects (hepatic, immunological, or haematological effects). However, NTB has been directly linked to melanoma replication, invasion, and migration via blockage of α4-integrin expressed in tumour cells, and to the development of melanoma in treated patients^21^.

Epidemiological studies on DMTs show data disparity^4^, although second-line immunosuppressants (azathioprine, cyclophosphamide, mitoxantrone) seem to involve a heightened risk for malignancy^22^. However, the relationship between immunotherapy and neoplasm development is inconclusive since some of these drugs are employed as coadjuvants or even anti-cancer drugs (ALB, cladribine, mitoxantrone) (http://www.ema.europa.eu), or are under consideration (FLM, TER) as oncologic therapeutic options^7,23^. This implies their subjection by Medicines Agencies to the monitoring of the possible development of malignancy in treated MS patients. For example, in the *safety analysis* recorded in the technical information, the overall incidence of cancer was twofold higher in cladribine-treated patients compared to patients who received a placebo (http://www.ema.europa.eu)^24^. It should be also highlighted that our patients will use them long-term.

Moreover, we found in our sample that TIFN and TDMF protected against neoplasm development, although INF and GA have been linked to cancer development^9,25–27^. However, a regressive effect of IFN has been casually observed in isolated neoplasms^28^, as well as a 32% lower mortality related to untreated patients^29^. Regarding DMF, which was recently licensed, a few studies have been published with a short follow-up, confirming its safe use (cancer was detected in only 0.9% of patients)^30^.

On the other hand, as we estimated (HR = 1.219, *p* = 0.077), a high risk could be expected from FLM use, supported by register-based cohort studies^31^, reviews^27^, or technical information: basal cell carcinoma being common (≥ 1/100– < 1/10), squamous cell carcinoma being uncommon (≥ 1/1000- < 1/100), Kaposi’s sarcoma or Merkel cell carcinoma, as well as cases of lymphoma being rare (≥ 1/10,000– < 1/1000) (http://www.ema.europa.eu).

SPMS acted as a protective factor for neoplasm development, but large retrospectives studies did not find differences in phenotype contribution with respect to a decrease in cancer risk^32^. A possible explanation for our finding could be the distinct cytokine and adhesion molecule expression pattern of MS variants^33^. This should be extensively examined. Likewise, the influence of the patient's disability status on the neoplasm development could be considered. The latter is a limitation of our study and, although we did not include this variable like many other papers, some authors have found a low disease process, according to the Expanded Disability Status Scale (EDSS)(≤ 2), in the majority of patients with benign tumours^15^.

Finally, to understand the extent of neoplasm development in MS patients, genetic predisposal factors and lifestyle must be explored, although some works observe a lack of information in this regard^22^. Multivariate analysis revealed smoking to be the most significant risk predictor for neoplasm/cancer. Female sex is a risk factor; however, it should be noted that women predominated in our study (19.4%, 13 of 66 had gynaecological neoplasms). Family predisposition was a moderate risk predictor. In fact, a comprehensive study showed that lower cancer risk in MS patients did not coincide with a lower risk in their parents^34^.

## Conclusions

In the patients studied, neoplasm prevalence was 23.4%, with a similar distribution of different types of tumours with respect to the general population, except for skin neoplasm (37.9% of occurring neoplasms). In our sample, 6.8% of patients suffered from cancer, in line with the data observed in other MS-focused studies. The extended Cox model identified smoking as the main risk factor for neoplasm development (HR = 3.938, CI 95% 1.392–11.140, *p* = 0.010), followed by the female gender (HR = 2.006, *p* = 0.030), and age at MS diagnosis (HR = 1.036, *p* = 0.04). SPMS (HR = 0.179, 0.042–0.764, *p* = 0.020) and treatment time with IFN (HR = 0.923, 0.873–0.977, *p* = 0.006) or DMF (HR = 0.725, *p* = 0.077) were protective factors. Tobacco and IFN treatment time lost their negative/positive influence as the result of an increase in survival time. The Cox regression model identified tobacco/AGE-DG a risk factor for cancer (HR = 1.099, CI95%1.001–1.208, *p* = 0.049), followed by FLM treatment time (HR = 1.219, *p* = 0.077).

In summary, genetic factor, lifestyle, the inflammatory profile of MS, drug type, and clinical practice interact in a complex manner. The last drugs introduced would require more clinical experience. Perhaps, exposure time of these risk factors should be taken into account, given the long-term nature of the disease.
